# A training package for zone III Resuscitative Endovascular Balloon Occlusion of the Aorta (REBOA)

**DOI:** 10.1186/1757-7241-22-S1-P18

**Published:** 2014-07-07

**Authors:** Robbie A Lendrum, Zane B Perkins, Gareth E Davies

**Affiliations:** 1 London’s Air Ambulance, The Royal London Hospital, London, UK; 2 Centre for Trauma Sciences, Queen Mary, University of London, UK

## Background

Non-compressible haemorrhage is the leading cause of preventable trauma death, with pelvic and groin haemorrhage associated with mortality rates approaching 50% [1,2]. Trauma systems expedite access to haemorrhage control, however, the majority of patients who die, exsanguinate before control can be achieved. REBOA is an innovative technique that provides the opportunity for meaningful improvements in the outcome of these patients. It involves the positioning of a balloon at the aortic bifurcation (Zone III) as a means of temporary in-flow control and afterload augmentation in patients with severe distal haemorrhage [3]. Our aim is to describe the training package developed to introduce zone III REBOA at a UK Major Trauma Centre.

## Methods

A multidisciplinary working group, consisting of consultants in Pre-Hospital Care, Emergency Medicine, Interventional Radiology, Anaesthesia and Trauma and Vascular Surgery, reviewed the existing REBOA literature and developed a training package that enables procedural knowledge and competence in potential operators.

## Results

The REBOA training package components are:

### 1) Required reading

Consists of six publications that detail the background, benefits, technical considerations and worldwide experience with the procedure.

### 2) Standard operating procedure (SOP)

An evidence-based SOP defines the target population, equipment, procedure and post-procedure management.

### 3) Written assessment

A written assessment, based on the required reading and SOP, tests trainees’ knowledge and understanding of the procedure.

### 4) Equipment familiarisation

A purpose built mannequin and complete set of training equipment allow trainees to gain technical familiarity with procedural steps and kit.

### 5) Moulage

Scripted scenarios are used in multidisciplinary, high fidelity, training “moulages” to test the trainees’ leadership, decision-making, teamwork and procedural competence.

### 6) “Sign-off”

Potential operators are required to successfully complete all components of the training package under the supervision of a “signed-off” operator.

## Conclusion

REBOA is less invasive and more effective at temporarily controlling exsanguinating pelvic haemorrhage than thoracotomy with aortic compression. As with resuscitative thoracotomy, many clinicians faced with a patient who would benefit from the procedure, will have no prior experience with REBOA. Our training package attempts to address this.
